# Identification of pheromone components and their binding affinity to the odorant binding protein CcapOBP83a-2 of the Mediterranean fruit fly, *Ceratitis capitata*

**DOI:** 10.1016/j.ibmb.2014.02.005

**Published:** 2014-05

**Authors:** P. Siciliano, X.L. He, C. Woodcock, J.A. Pickett, L.M. Field, M.A. Birkett, B. Kalinova, L.M. Gomulski, F. Scolari, G. Gasperi, A.R. Malacrida, J.J. Zhou

**Affiliations:** a Department of Biological Chemistry and Crop Protection, Rothamsted Research, Harpenden, Herts. AL5 2JQ, United Kingdom; b Dipartimento di Biologia e Biotecnologie, Università di Pavia, Via Ferrata 9, 27100 Pavia, Italia; c Institute of Organic Chemistry and Biochemistry of the AS CR, v.v.i., Flemingovo nám. 2, CZ-166 10 Prague 6, Czech Republic

**Keywords:** Medfly, *Ceratitis capitata*, Olfaction, Odorant binding protein, Pheromone binding protein, Pheromone, Binding studies, Protein expression, Electroantennography, GC–EAG, Fluorescence displacement, OBP, Odorant binding protein, PBP, Pheromone binding protein, CSP, Chemosensory protein, OR, Odorant receptor, EAG, Electroantennography, GC–EAG, Gas chromatography coupled to electroantennography, SIT, Sterile Insect Technique

## Abstract

The Mediterranean fruit fly (or medfly), *Ceratitis capitata* (Wiedemann; Diptera: Tephritidae), is a serious pest of agriculture worldwide, displaying a very wide larval host range with more than 250 different species of fruit and vegetables. Olfaction plays a key role in the invasive potential of this species. Unfortunately, the pheromone communication system of the medfly is complex and still not well established. In this study, we report the isolation of chemicals emitted by sexually mature individuals during the “calling” period and the electrophysiological responses that these compounds elicit on the antennae of male and female flies. Fifteen compounds with electrophysiological activity were isolated and identified in male emissions by gas chromatography coupled to electroantennography (GC–EAG). Within the group of 15 identified compounds, 11 elicited a response in antennae of both sexes, whilst 4 elicited a response only in female antennae. The binding affinity of these compounds, plus 4 additional compounds known to be behaviourally active from other studies, was measured using *C. capitata* OBP, CcapOBP83a-2. This OBP has a high homology to *Drosophila melanogaster* OBPs OS-E and OS-F, which are associated with trichoid sensilla and co-expressed with the well-studied *Drosophila* pheromone binding protein LUSH. The results provide evidence of involvement of CcapOBP83a-2 in the medfly's odorant perception and its wider specificity for (*E*,*E)*-α-farnesene, one of the five major compounds in medfly male pheromone emission. This represents the first step in the clarification of the *C. capitata* and pheromone reception pathway, and a starting point for further studies aimed towards the creation of new powerful attractants or repellents applicable in the actual control strategies.

## Highlights

• The pheromone blend produced by medfly males during the calling period is investigated using air entrainment and GC–EAG.
• Out of 15 chemicals, 11 elicited antennal responses in both sexes and 4 elicited the response only in female antennae.
• The first medfly odorant binding protein CcapOBP83a-2 has been expressed and purified (87).
• CcapOBP83a-2 showed binding affinity for (*E*,*E*)-α-farnesene, one of the components of medfly pheromone.
• The ability of CcapOBP83a-2 to bind other electrophysiologically active compounds shows its broad binding specificity.

## Graphical abstract

**Figure:**
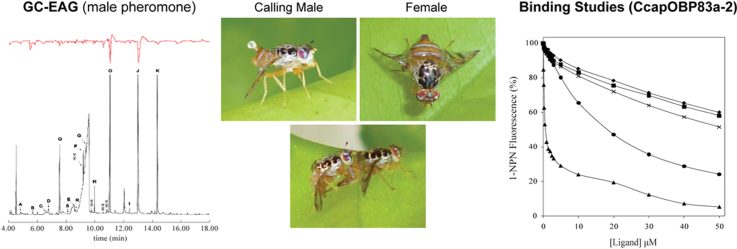

## 1 Introduction

Insect pheromones play an important role in intra- and inter-species communication, inducing specific behavioural responses in terms of sexual attraction, mating aggregation and host-marking of oviposition sites. Odour perception is regulated by a fine molecular pathway that involves multigene families including odorant-binding proteins (OBPs), chemosensory proteins (CSPs) and odorant receptors (ORs). Within the OBP family, pheromone-binding proteins (PBPs) are proven to be involved in insect sexual communication, but the molecular basis underlying this process is still unknown for most Dipteran insects and is the target of several studies. The Mediterranean fruit fly or medfly, *Ceratitis capitata* (Wiedemann; Diptera: Tephritidae) is a serious pest of agriculture worldwide (Arita and Kaneshiro, 1989; Maddison and Bartlett, 1989). This species shows a wide larval host range comprising more than 250 different species of fruits and vegetables (Joint-FAO/IAEA-Division, 1985; Malacrida et al., 2007), which is a major factor in its biological success. Olfaction plays a key role in the invasive potential of this species, regulating essential behaviours such as i) localisation of plant hosts, ii) detection of pheromones during recognition and location of mates for mating, iii) discrimination between suitable and already pierced hosts for oviposition. The pheromone communication system of the medfly is complex and still not fully understood. Medfly mating behaviour has been a topic of extensive research in the last few decades (Arita and Kaneshiro, 1989; Feron, 1959; Levinson et al., 1987; Prokopy and Hendrichs, 1979; Shelly et al., 1994; Whittier et al., 1992; Yuval and Hendrichs, 2000; for a review, see Eberhard, 2000). In this species the males attract females (Arita and Kaneshiro, 1989; Feron, 1959; Prokopy and Hendrichs, 1979) by emitting a mixture of pheromone compounds. Several decades ago Feron (1962) reported compounds released by *C. capitata* males. Since then, various studies have been conducted, with the aim of identifying the active components of the pheromone mixture (Alfaro et al., 2011; Feron, 1962; Goncalves et al., 2006; Jacobson et al., 1973; Jang et al., 1989; Ohinata et al., 1977). Jacobson and colleagues (Jacobson et al., 1973) described the sex pheromone as a mixture of 15 substances, including carboxylic acids and other compounds such as methyl (*E*)-6-nonenoate and (*E*)-6-nonen-1-ol. In 1977, Ohinata and colleagues reported that these mixtures were attractive to both sexes in laboratory tests, but only to males in an open field trial (Ohinata et al., 1977). Jang et al. (1989) detected 69 compounds from the male headspace, while the female headspace samples contained traces of only a few compounds, mainly short-chain aldehydes. Goncalves (Goncalves et al., 2006) published another list of compounds and reported the composition of aeration samples of calling males collected in Tenax tubes. More recently, Alfaro et al. (2011) reported the medfly volatile profiles at different physiological states and characterised groups of compounds according to their emission pattern. As the exact composition and function of the complex pheromone blend, and the molecular mechanisms by which it is sensed by both males and females, is not well defined, further studies on these topics may furnish powerful tools for the improvement of current pest control strategies, i.e. by developing specific synthetic attractants or repellents that can be used in Sterile Insect Technique (SIT) programmes.

The analysis of medfly EST libraries and the genome sequence resulted in the identification of a number of *obp* genes (Gomulski et al., 2012, 2008; Scolari et al., 2012; Siciliano et al., 2014). Further molecular characterisation and analysis of expression profiles of five identified putative *obp* genes (*CcapOBP69a*, *CcapOBP19d-1*, *CcapOBP83a-1*, *CcapOBP83a-2* and *CcapOBP28a*) underlined a possible implication of these genes in odorant perception and represented a first step in the elucidation of the molecular pathway regulating olfactory behaviours in the medfly (Siciliano et al., 2014). One of these five genes, *CcapOBP83a-2,* was found to be highly enriched in antennae with the highest expression in sexually mature individuals (Siciliano et al., 2014). In a phylogenetic analysis (Siciliano et al., 2014) it is clustered together with *Drosophila melanogaster* OBPs OS-E and OS-F also known as OBP83a and OBP83b that have been shown to associate with trichoid sensilla, which are strongly implicated in the detection of volatile pheromones. OS-E and OS-F were identified by McKenna and colleagues (McKenna et al., 1994). OS-F was also independently and simultaneously identified as PBPRP3 by Pikielny et al. (1994). OS-E and OS-F are known to co-express with a well-studied *Drosophila* OBP called LUSH in the pheromone sensitive sensilla (Shanbhag et al., 2001); while ligands are known for LUSH as 11-*cis*-vaccenyl acetate (cVA) (Ha and Smith, 2006), no ligands are known for OS-E and OS-F. Interestingly the analyses of medfly EST libraries and the genome sequence identified a LUSH-like OBP gene, *CcapOBP19a,* in the medfly genome (Siciliano et al., 2014). In the present study, we investigate the chemicals emitted by sexually mature medfly individuals during the “calling” period using coupled gas chromatography–electroantennography (GC–EAG) and coupled GC–mass spectrometry (GC–MS) in order to identify the physiologically active compounds. We then expressed the first *C. capitata* OBP CcapOBP83a-2 and used it to examine the relative binding ability of the newly identified pheromone components. We provide evidence for the possible involvement of CcapOBP83a-2 in olfaction processes (possibly in the pheromone communication) and its specificity for (*E*,*E*)-α-farnesene, one of the five major compounds in the medfly male pheromone emission.

## 2 Material and methods

### 2.1 Collection of volatiles

Medfly individuals were obtained from the well-established ISPRA strain and reared under standard conditions in the quarantine facility at the University of Pavia, Italy. Volatile collections were performed over 24 h (from 15:00 h to 15:00 h) using 4 day-old virgin flies (60 flies per vessel) from males and females separately. Each set of 60 individuals (2 replicates per sex) was placed inside an airtight 2-L glass vessel connected to the outlet of an air compressor which pumped air through a charcoal filter guaranteeing the use of ultrapure air (550 ml/min). Volatiles were collected on Porapak Q (0.05 g, 60/80 mesh; Supelco) in a glass tube (5 mm Ø) inserted into the collection port on top of the vessel. Another pump drew air through this tube. To ensure that no unfiltered air was drawn into the vessel from outside, the rates of airflow were set so that more purified air was pumped in than was drawn out. After 24 h, volatiles were eluted from the Porapak Q tube with 0.5 ml of redistilled diethyl ether, providing a solution that contained the isolated volatile compounds. Samples were then stored at −20 °C until used. Before use, all the equipment was rinsed with acetone, ethanol and distilled water and then dried in an oven at 180 °C for at least 2 h. Porapak Q tubes were cleaned by elution with redistilled diethyl ether and heated at 132 °C for 2 h under a stream of purified nitrogen to remove contaminants. Charcoal filters were conditioned before use by attaching to a constant stream of nitrogen in an oven at 150 °C for 2 h.

### 2.2 Gas chromatography (GC) analysis

GC analysis was performed by injecting 4 μl of volatile sample onto a nonpolar capillary column (HP-1, 50 m, 0.32 mm internal diameter, 0.52 μm film thickness) using an Agilent 6890 GC equipped with a cold on-column injector and flame ionization detector (FID). The oven was maintained at 30 °C for 2 min and then programmed for increments at 5 °C min^−1^ to 250 °C. Quantification was carried out by calculating and comparing peak areas with known amounts of authentic external standards.

### 2.3 Coupled gas chromatography–mass spectrometry (GC–MS) analysis

Attractive headspace samples were analysed on a capillary GC column (HP-1, 50 m, 0.32 mm i.d., 0.52 μm f.t.) directly coupled to a mass spectrometer (VG Autospec, Fisons Instruments, Manchester, UK) equipped with a cold on-column injector. Ionization was initiated by electron impact (70 eV, 250 °C). The oven was maintained at 30 °C for 2 min and then programmed for increments at 5 °C min^−1^ to 250 °C. Tentative GC–MS identifications were confirmed by peak enhancement with authentic standards on two GC columns of different polarities (Ukeh et al., 2009).

### 2.4 Coupled gas chromatography–electroantennography

Electrical responses to chemical stimuli were recorded from the antennae of both sexes using 4 day-old virgin individuals. Electroantennogram (EAG) recordings were made with Ag–AgCl glass microelectrodes filled with Ringer solution (for 1 L H_2_O solution: 7.2 g NaCl, 0.37 g KCl, 0.17 g CaCl_2_, pH 7.3–7.4). The head of a fly (both male and female), anaesthetised by chilling, was excised from the body with a microscalpel and the tip of one of the two arista was cut off with micro-scissors. The indifferent electrode was inserted in the head (from the base to the top-frontal part) and the recording electrode was positioned over the cut arista. Signals were passed through a high impedance amplifier (UN-06, Hilversum, The Netherlands) and analysed using a customised software package (Syntech). The coupled GC–EAG system, in which the effluent from the GC column is simultaneously directed to the antennal preparation and the GC detector, has been described by Wadhams (1990). Separation of volatiles was achieved on a GC (Agilent Technologies, 6890N) equipped with a cold on-column injector and a flame ionization detector (FID) using an HP-1 column (50 m, 0.32 mm i.d., 0.52 μm f.t.). The oven was maintained at 30 °C for 2 min and then programmed for increments at 5 °C min^−1^ to 250 °C. The carrier gas was helium. Outputs from the EAG amplifier and the FID were monitored simultaneously and analysed using the Syntech software package. Peaks eluting from the GC column were considered active if they elicited EAG response in three or more coupled runs. Authentic standards were tested using a delivery system which employed a filter paper strip in a disposable Pasteur pipette cartridge (Wadhams et al., 1982). The stimulus (2 s duration) was delivered into a purified airstream (1 l/min) flowing continuously over the preparation using a stimulus controller (Syntech CS02). Samples (10 μl) of the standard solution of test compounds (1 mg/ml in redistilled hexane) were applied in the cartridge. The control stimulus was hexane (10 μl). Five replicates were performed for each sample using antennae from 5 different flies.

### 2.5 Phylogenetic analysis

A phylogenetic analysis was performed including 17 medfly OBP amino acid sequences (Siciliano et al., 2014), the 52 known *D. melanogaster* OBPs (Hekmat-Scafe et al., 2002; Vieira and Rozas, 2011; Zhou et al., 2008) and OBPs from three other tephritid species, *Bactrocera dorsalis s.s.* (Zheng et al., 2013), *Rhagoletis suavis* (Ramsdell et al., 2010), and *Rhagoletis pomonella* (Schwarz et al., 2009). After excluding the signal peptide sequence, the OBP amino acid sequences were aligned using MAFFT v6.935b (Katoh et al., 2005) with the E-INS-i strategy, BLOSUM62 matrix, 1000 maxiterate and offset 0. Phylogenetic relationships were estimated using Maximum Likelihood with 1000 bootstrap replications using MEGA 5.2.2 (Tamura et al., 2011).

### 2.6 Intron/exon structure

The genomic and CDS sequences of *Drosophila* genes were downloaded from the Flybase (http://flybase.org/). The genomic and cDNA sequences of the medfly OBP genes CcapObp83a-1 and CcapObp83a-2 were downloaded from NCBI website with the accession numbers of XM_004523388.1 and XM_004523387.1, respectively. The genomic sequences were compared and aligned with CDS sequences manually. The size and number of exons and introns of each gene were counted.

### 2.7 Cloning and sequencing

Antennal cDNA was subjected to PCR using primers designed based on the gene sequence deposited in GenBank (accession No. XM_004523387) for cloning of the fragment encoding the mature CcapOBP83a-2 (without signal peptide, see Figure S1), flanked by Nhe*I* and Hind*III* restriction sites at the 5′-end and 3′-end respectively (CcP4NheI: ataGCTAGCCAAAAGGAGTTAAGACG, CcP4HindIII: gcgAAGCTTTCAAATCAAGAAATA). The PCR product with the correct size was separated on 1% agarose gel and then excised and purified with the Wizard^®^ SV Gel and PCR Clean-Up System (Promega) following the manufacturer's instructions. The purified PCR product was ligated into pGEM^®^-T Easy vector (Promega) using a 1:4 M ratio (vector: PCR product) by incubating the mixture with T4-DNA ligase and T4 ligase buffer at room temperature for 3 h. The ligation reaction mix (5 μl) was used to transform 50 μl of TOP10 *Escherichia coli* competent cells (Invitrogen). Positive colonies were selected by their ampicillin resistance, white/blue screening and PCR with gene specific primers. Plasmid DNA containing CcapOBP83a-2 coding region from positive white colonies was extracted using the Wizard^®^ *Plus* SV Minipreps DNA Purification System (Promega) and then sequenced. The derived protein sequence of CcapOBP83a-2 was compared and aligned with other members of insect OBPs using software SIAS (http://imed.med.ucm.es/Tools/sias.html) with default settings for identity and similarity calculations.

### 2.8 Subcloning in expression vector pET17b

The purified construct pGEM-CcapOBP83a-2 was double digested with Nhe*I* and Hind*III* restriction enzymes at 37 °C overnight. Digested product was loaded on 1% agarose gel, purified and ligated into pET17b (Novagen) previously linearized with the same enzymes. 5 μl of ligation were used to transform TOP10 cells and purified DNA from positive clones (as above) was sequenced to verify the right position and orientation of the inserted gene. Sequence analyses were performed using CLC Main Workbench software (CLC bio).

### 2.9 Bacterial expression of CcapOBP83a-2

To express recombinant protein CcapOBP83a-2, *E. coli* BL21(DE3)pLysS cells were transformed with the construct pET17b-CcapOBP83a-2. A positive colony was selected as described above and used to inoculate 5 ml of fresh LB/ampicillin medium at 37 °C overnight, and scaled up with 1 L of fresh LB/ampicillin medium. Protein expression was induced for 3 h by adding IPTG (to a final concentration of 0.4 mM) when the culture had reached an O.D_600_ value of 0.7–0.9. Cells were then harvested by centrifugation at 3000 × *g* and lysed by sonication. The expressed CcapOBP83a-2, present as inclusion bodies, was solubilised in 3 ml of 5 M urea, 25 mM DTT (in 20 mM Tris pH 7.4) and then treated with 250 μl of 100 mM cystine (in 0.5 M NaOH) and 5 ml of 5 mM cysteine (in 100 mM Tris–HCl pH 10). The solution was shaken at 28 °C overnight and then dialysed against 20 mM Tris–HCl pH 7.4. CcapOBP83a-2 was then purified by a first round of anion-exchange chromatography with a HiPrep 16/40 column (GE Healthcare, Hatfield, UK) filled with a DE-52 resin (Whatman, Kent, UK) followed by gel filtration on a Sephacryl S-200 HiPrep 26/60 column with an ÄKTA FPLC system (GE Healthcare, Hatfield, UK). The fraction collections obtained were concentrated by using a vacuum drier, quantified by Nanodrop (Thermo Scientific), delipidated at pH 4.5 with 100 μl of Lipidex-1000 (PerkinElmer Inc.) for 1 h and then re-folded by dialysis against 20 mM Tris–HCl pH 7.4 at 4 °C. All purification steps were monitored by SDS-PAGE. The final purified proteins were also tested on both SDS-PAGE and native-PAGE (without SDS or β-mercaptoethanol in order to avoid protein denaturation). Four different protein amounts (10 μg, 5 μg, 1 μg and 0.1 μg) were loaded into individual lane on the native-PAGE gel.

### 2.10 Fluorescence displacement binding assay

To measure the binding affinity of the fluorescent probe 1-NPN to CcapOBP83a-2, a 2 μM solution of protein in 20 mM Tris–HCl pH 7.4 was titrated with aliquots of 1 mM 1-NPN stock to a final concentration of 0.1–50 μM in methanol. The 1-NPN/protein mixture was excited at 337 nm and the emission was recorded between 380 and 450 nm on a Perkin Elmer LS 55 Fluorescence spectrometer (Cambridge–UK) at 25 °C in a right angle configuration with a 1 cm light path quartz cuvette. The excitation and emission slits were both 5 nm. Data were recorded and graphed using FL WinLab Software (Perkin–Elmer). The affinity of other ligands was measured in competitive binding assays, using 1-NPN as fluorescent reporter at 3 μM concentration and final concentrations of each competitor from 0.1 μM to 50 μM. To determine binding constants, the fluorescent intensity values at the maximum emission (after subtracting those measured without protein) were plotted against free ligand concentrations. The level of bound-ligand was evaluated from the value of fluorescence intensity, assuming that the CcapOBP83a-2 had a 100% activity with a stoichiometry of 1:1 protein:ligand at saturation. IC_50_ values (the concentration of ligand halving the initial fluorescence level) were used to calculate each competitor dissociation constants (*K*_*D*_), by the equation: *K*_*D*_ = IC_50_/1 + [1 − NPN]/*K*_1-NPN_, where [1 − NPN] is the free concentration of 1 − NPN and *K*_1-NPN_ is the dissociation constant of the 1-NPN/protein complex. Analyses were performed by nonlinear regression curve fitting using GraphPad Prism 5 (GraphPad Software, Inc, La Jolla, USA).

### 2.11 Chemicals

3-Methylbutan-1-ol (≥99%), (*RR*)-2,3-butanediol (98%), (*RS*)-3-methyl-2-pentanone (99%), (*R*S)-2-methylbutyric acid (≥98%), myrcene (≥90%), indole (≥99%), geranyl acetate (98%), dihydro-3-methyl-2-(3H)-furanone (100%), ethyl (*E*)-3-hexenoate (100%), tetrahydro-3,4-furandiol (95%) and methyleugenol (≥98%) were purchased from Sigma Aldrich. (*RS*)-Linalool was purchased from Avocado Research Chemicals. (*Z*)-Ocimene (70%) was purchased by Bush-Boake Allen. (*E*)-ocimene (≥95%), *E*-β-farnesene (≥98%) and (*E*,*E*)-α-farnesene (≥95%) were synthesised in the BCCP Department at Rothamsted Research, UK. Trimedlure (100%) was purchased from Farma Tech International Corp. Ethyl (*E*)-3-octenoate (100%) and ethyl octanoate (100%) were synthesised by Dr. Michal Hoskovec, Institute of Organic Chemistry and Biochemistry, Czech Republic.

## 3 Results & discussion

### 3.1 Identification of compounds that elicited an EAG response

**Fig. 1:**
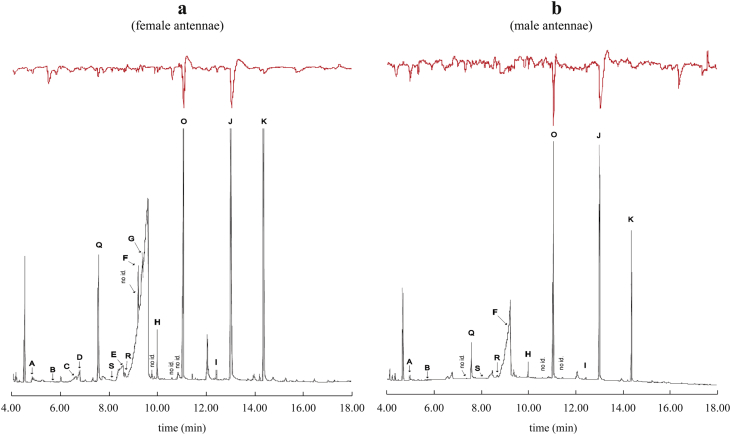
Typical GC–EAG recordings of the male extract. Red lines represent electrophysiological responses on antennae of females (a) and males (b). Compounds are labelled by letters. Respective chemical names are listed in Table 1. (For interpretation of the references to colour in this figure legend, the reader is referred to the web version of this article.)

Coupled GC–EAG analyses revealed that 15 compounds in the male volatile extract elicited an electrophysiological response on female and male antennae of *C. capitata* (labelled as A–K, O, Q, R and S in Fig. 1 and chemical names listed in Table 1), including 5 major compounds (Q, F, O, J and K) reported for the medfly, consistent with a previous GC–EAG report using pure chemicals (Jang et al., 1989). Tetrahydro-3,4-furandiol (S) is a newly identified compound. The other fourteen compounds have already been reported in previous studies, including (*E*,*E*)-α-farnesene which elicited small responses in both male and female flies (Fig. 1). Four of 15 compounds [(*RS*)-3-methyl-2-pentanone, (*RS*)-2-methylbutyric acid, myrcene and (*E*)-ocimene] elicited a response only on the female antennae (Table 1). These results suggest that there is a difference in the pheromone perception between male and female medflies. Moreover, GC–EAG data showed that most female antennae responded to both (*E*)-ocimene (M0F4) and (*Z*)-ocimene (M4F4), while male antennae only respond to (*Z*)-ocimene (M4F4) (Table 1). This difference in the electrophysiological response to the two ocimene isomers indicates that the medfly olfactory systems may be able to discriminate between different isomers of the same chemical. This ability can be due to the different activity or presence of specific OBPs, ORs or both between male and female antennae. The GC–EAG recordings also revealed that the medfly strain, reared in laboratory conditions for generations and used in this study, still conserves the innate sensitivity to the pheromone blend components (Jang et al., 1989; Vaníčková et al., 2012).

### 3.2 CcapOBP83a-2, an OS-E/OS-F homologue enriched in the medfly antennae

**Fig. 2:**
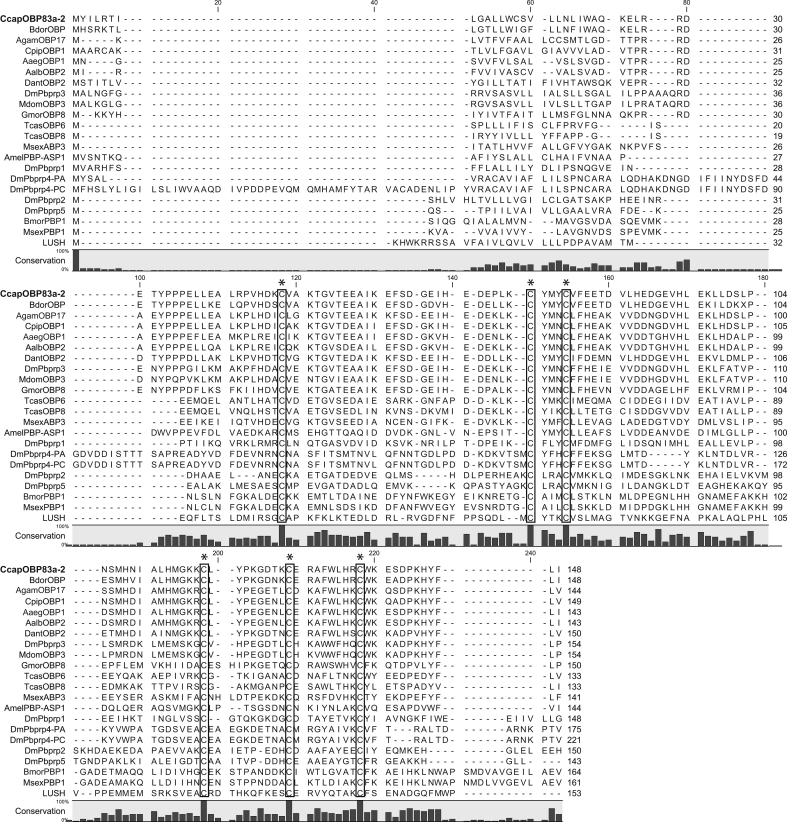
Alignment between CcapOBP83a-2 and other known insect PBP/OBP amino acid sequences. Bdor (*Bactrocera dorsalis*), Agam (*Anopheles gambiae*), Cpip (*Culex pipiens quinquefasciatus*), Aaeg (*Aedes aegypti*), Aalb (*Aedes albopictus*), Dant (*Delia antiqua*), Dm (*Drosophila melanogaster*), Mdom (*Musca domestica*), Gmor (*Glossina morsitans*), Tcas (*Tribolium castaneum*), Msex (*Manduca sexta*), Amel (*Apis mellifera*), Bmor (*Bombyx mori*). Conserved cysteines are boxed. The black bars below the alignment indicate the conservation as percentage of an amino acid residue relative to total number of residues at the same position.

The predicted protein sequence of the cloned *CcapObp83a-2* gene displayed all the structural features typical of insect OBPs, i.e. i) the presence of 6 cysteines in highly conserved positions, typical of insect “Classic OBP” subfamily, ii) a good level of similarity with putative PBPs of other insect species (Table S1), iii) the presence of a signal peptide of expected size (between 20 and 35 aa) at the N-terminal, iv) a protein size of 120–150 amino acids. The *CcapObp83a-2* sequence and protein translation are reported in Fig. 2 and Supplementary Fig. S1 and available in GenBank with the accession number XM_004523387. Expression profile analyses by real time quantitative RT-PCR have shown that the transcription of *CcapObp83a-2* gene is enriched in antennae and increases in relation to sexual maturation, while the transcript of *CcapObp83a-1* is also highly expressed in the palps (Siciliano et al., 2014). In Drosophila, *OS-F* (*OBP83a)* gene is also expressed predominantly in the antennae (Hekmat-Scafe et al., 2000; McKenna et al., 1994). The size and number of exons and introns of these genes are listed in Table 2. Both *CcapObp83a-2* and *CcapObp83a-1* have similar intron/exon structures as *OS-F*, while the intron 2 of *CcapObp83a-1* is much longer than those of the other genes. The OS-E and OS-F genes are adjacent (Hekmat-Scafe et al., 1997) and strongly supported the product of gene duplication in *D. melanogaster* (Hekmat-Scafe et al., 2000). *CcapObp83a-1* and *CcapObp83a-2* are also adjacent on scaffold NW_004523725.1 (data not shown) and thus may also represent duplicated genes as in *D. melanogaster*, *Drosophila teissieri*, *Drosophila willistoni* and *Drosophila lebanonensis* but unlike *Drosophila simulans*, *Drosophila mauritiana* and *Drosophila virilis* where only one of these genes is present (Hekmat-Scafe et al., 2000). This suggests that the OS-E/OS-F gene duplication may precede the common ancestor of medfly and *Drosophila*. It has been suggested that these two genes underwent divergent evolution under positive selection leading to the functional diversification of new copies (Sanchez-Gracia and Rozas, 2008).

**Fig. 3:**
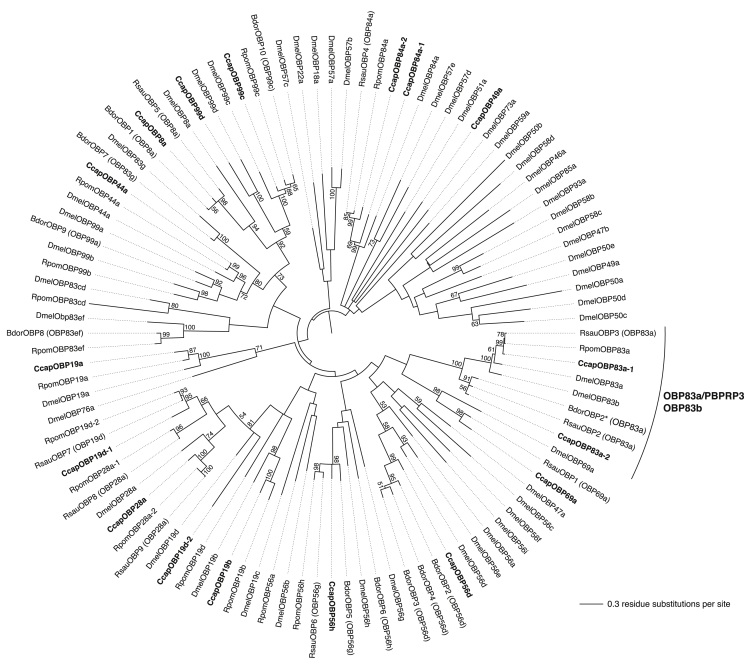
Phylogenetic relationships of medfly, *Drosophila melanogaster*, *Bactrocera dorsalis*, *Rhagoletis suavis* and *R. pomonella* OBP proteins. Mid-point rooted maximum-likelihood (log likelihood = −20855.92) tree inferred using the Whelan and Goldman model (Whelan and Goldman 2001) and a discrete Gamma distribution and some invariable sites. Bootstrap values greater than 50% (1000 replications) are shown. OBP nomenclature follows that of Siciliano et al. (2014).

We further investigated the relationships between the *Drosophila* OS-E/OS-F proteins with other OBPs from the medfly, *D. melanogaster*, *B. dorsalis*, *R. suavis* and *R. pomonella* using a phylogenetic approach. In the mid-point rooted Maximum Likelihood tree (Fig. 3) a well-defined cluster supported by a 100% bootstrap comprising of OBP83a (OS-E) and OBP83b (OS-F) and their homologues in the other species. CcapOBP83a-1 is tightly clustered in a sub-cluster with both OBP83a and OBP83b, but shares higher identity/similarity to OBP83a (78/89%). CcapOBP83a-2 falls into another sub-cluster with two homologues from *B. dorsalis* and *R. suavis*, and shares higher identity/similarity with OBP83a (61/78%) than OBP83b (54/77%) (Fig. 3), suggesting a functional divergence in the medfly (Sanchez-Gracia and Rozas, 2008; Vieira and Rozas, 2011).

### 3.3 CcapOBP83a-2 heterologous expression and purification

**Fig. 4:**
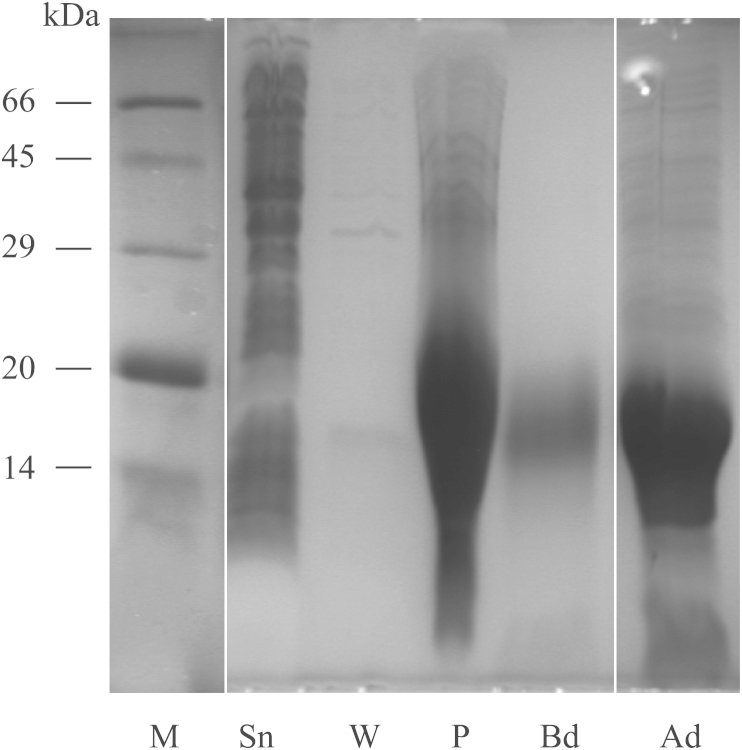
CcapOBP83a-2 protein purification. Recombinant CcapOBP83a-2 is not secreted in the extracellular medium (lane W), but is stored as inclusion bodies (lane P). Sn: culture after sonication; W: washing (supernatant); P: pellet; Bd: culture before dialysis; Ad: culture after dialysis; M: protein weight marker (BSA: 66 kDa, Ovalbumin: 45 kDa, Carbonic anhydrase: 29 kDa, Trypsin inhibitor: 20 kDa, Lactalbumin: 14 kDa).

**Fig. 5:**
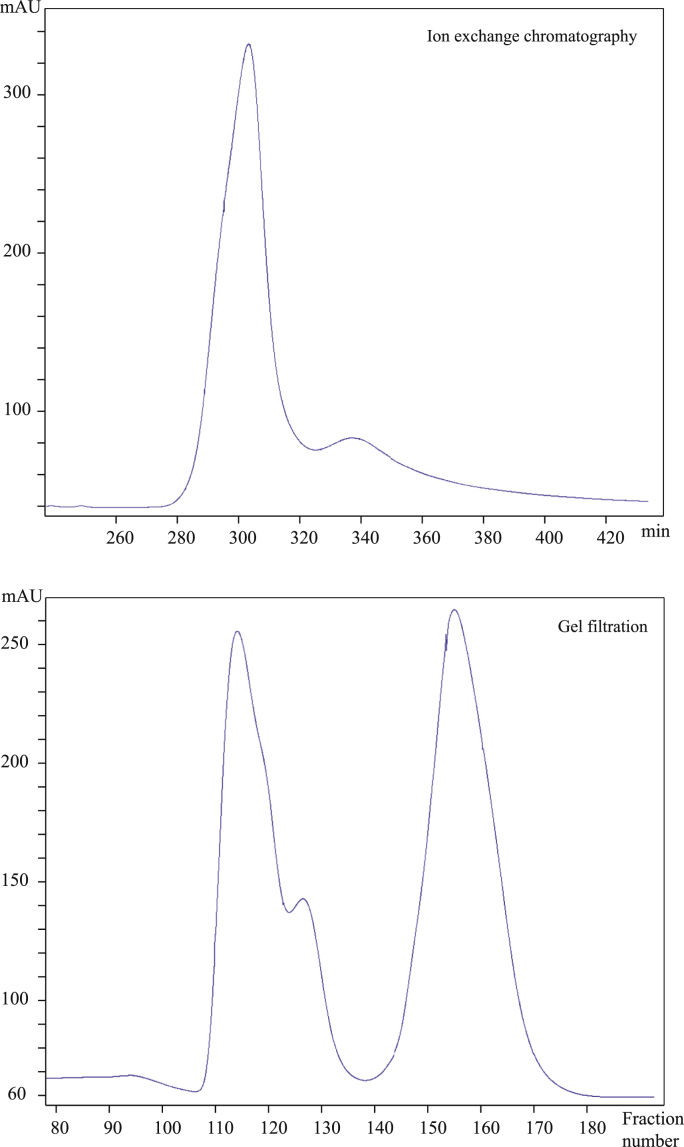
CcapOBP83a-2 purification through chromatography. Although only one peak (one peptide) was identified after ion exchange chromatography (upper panel), gel filtration revealed that two different proteins with different size were present in solution (lower panel). Values are reported in mAU (milli Absorbance Unit) on minutes (upper panel) and on fraction number (lower panel).

**Fig. 6:**
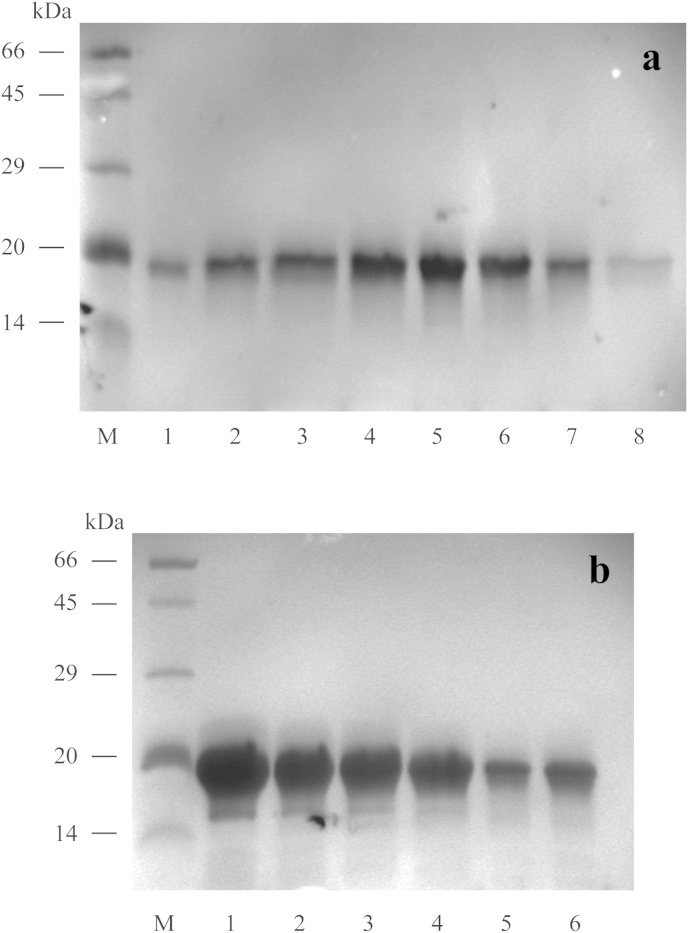
SDS-PAGE of purified protein fractions. The fractions related to each peak (Fig. 4, lower panel) were loaded on SDS-PAGE for the first peak related fractions (**a**) and the second peak related fractions (**b**). No differences in relation to the molecular weight were identifiable through under denature condition. M: weight marker (BSA: 66 kDa, Ovalbumin: 45 kDa, Carbonic anhydrase: 29 kDa, Trypsin inhibitor: 20 kDa, Lactalbumin: 14 kDa).

**Fig. 7:**
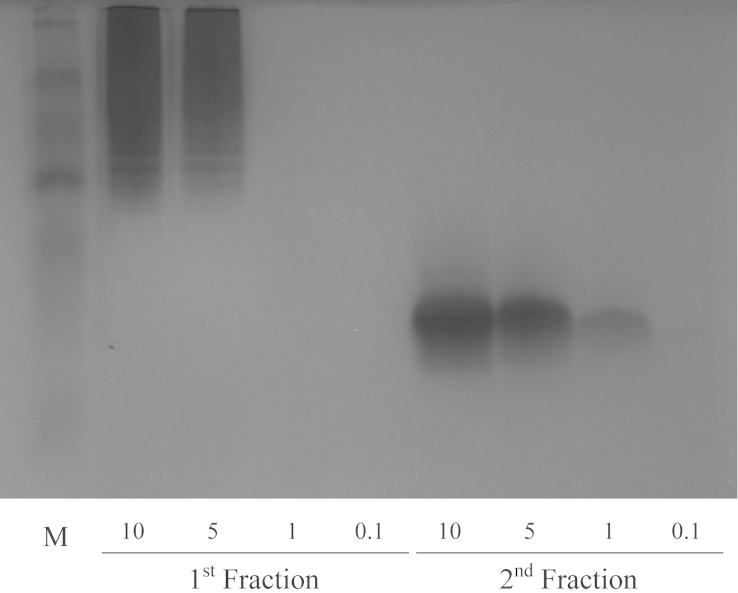
Polyacrylamide gel electrophoresis (PAGE) under native conditions. The purified protein from two fractions were loaded on a polyacrylamide gel in native conditions (without SDS and β-mercaptoethanol). Four different concentrations (10 μg, 5 μg, 1 μg and 0.1 μg in total) were loaded. The marker (M) is not clearly visible due to the electrophoresis un-denaturing conditions. The smear related to the first fraction may represent protein aggregating and incorrect refolding after the cleaning steps.

Expression of CcapOBP83a-2 in *E. coli* yielded a high amount of protein (≈2 mg/ml), which was present as insoluble inclusion bodies (Fig. 4). It was unfolded, purified and re-folded by dialysis (data not shown). Purification was achieved by using conventional ion-exchange and gel filtration chromatography. As shown in Fig. 5, we obtained only one peak from the first purification step by ion exchange chromatography, while two different peaks were present during gel filtration, indicating a good purity for the recombinant protein, but two forms that differed in size. The two fractions were then treated separately. Electrophoretic analyses showed that the proteins from these two fractions had the same molecular weight in denatured conditions (SDS-PAGE) (Fig. 6), but they run differently in native conditions (Fig. 7).

### 3.4 Fluorescent displacement binding assay

**Fig. 8:**
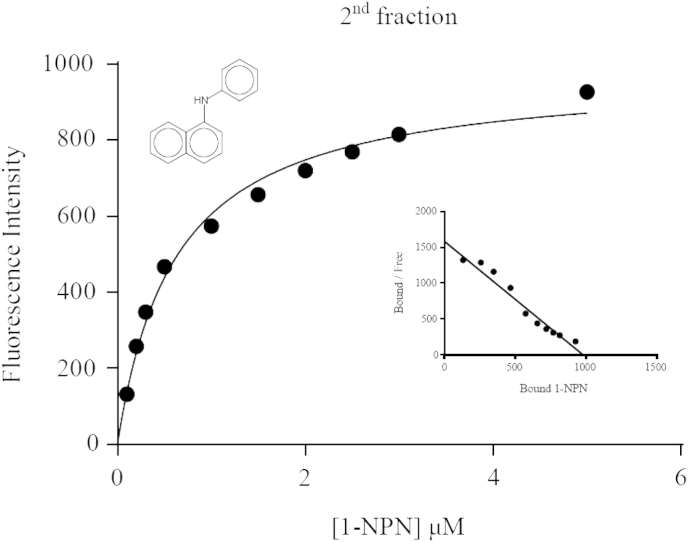
Binding of 1-NPN to CcapOBP83a-2. The purified CcapOBP83a-2 was diluted in 20 mM Tris–HCl pH 7.4 with a final concentration of 2 μM. Aliquots of 1-NPN stock solution [1 mM] in methanol were added to a final concentration of 0.1, 0.2, 0.3, 0.5, 1, 1.5, 2, 2.5, 3, 5 and 10 μM. Excitation was set at 337 nm, the peak emission at 380–460 nm was recorded and plotted against 1-NPN concentrations. The curve was used to determine the dissociation constant (*K*_*D*_ 2nd fraction: 0.617 ± 0.069 μM) by nonlinear regression curve fitting using GraphPad Prism 5, and transformed to Scatchard plot (insert).

We then measured the interactions between the medfly OBP CcapOBP83a-2 and electrophysiologically active compounds identified in the pheromone mixture emitted by *C. capitata* males during “calling”, as the first step for the molecular characterization of the OS-E/OS-F cluster, whose ligands have not been identified. The fluorescent dye displacement assay was employed as the competitive binding assay. In recent years, this particular method has been utilised in many studies on OBP binding activities and has been demonstrated to be effective and reliable (Gong et al., 2009; Guo et al., 2012; He et al., 2010; Qiao et al., 2011; Spinelli et al., 2012; Sun et al., 2013; Vandermoten et al., 2011; Yang et al., 2011; Zhang et al., 2012). The technique is based on the usage of the fluorescent probe N-phenyl-1-naphthylamine, 1-NPN, a lipophilic crystalline solid with the peculiarity of having a strong ability to bind insect OBPs (Ban et al., 2003). In previous studies (Lagarde et al., 2011) it was found that after heterologous expression some insect PBPs had fatty acid chains entrapped in their binding pockets. The presence of these molecules within the globular PBP structure could cause a spatial obstruction around the binding site, resulting in structural changes and different binding affinities for the protein to possible ligands. This drawback could lead to aberrant and untruthful results in the subsequent binding assays, thus a “cleaning” step of the purified CcapOBP83a-2 fractions by delipidation with Lipidex 100 (Perkin Emer, UK) was carried out. The two delipidated CcapOBP83a-2 fractions displayed a different affinity for the fluorescent probe 1-NPN, showing saturation levels in the range of 10–20 μM and 5–10 μM, respectively, at the protein concentration of ≈2 μM and dissociation constants (*K*_*D*_) of 10.5 ± 1.4 μM and 0.6 ± 0.1 μM for the first and second fraction, respectively (Fig. 8 and Supplementary Fig. S2).

For our binding studies, the 15 electrophysiologically active compounds identified in male pheromone emissions were tested for their binding ability to displace 1-NPN from the newly identified medfly OBP CcapOBP83a-2. Additionally, three chemicals demonstrated to be electrophysiologically and/or behaviourally active on *C. capitata* (Trimedlure, ethyl octanoate and methyleugenol) and (*E*)-β-farnesene [structurally related to (*E,E*)-α-farnesene] were also tested. The first fraction showed very poor binding activity with almost all of the chemicals tested (Figs. S3 and S4), and a limited degree of binding only with (*E*)-β-farnesene, with a low Ki value of 1.4 μM (Table S2), indicating that the first fraction might have aggregated and was not refolded properly.

**Fig. 9:**
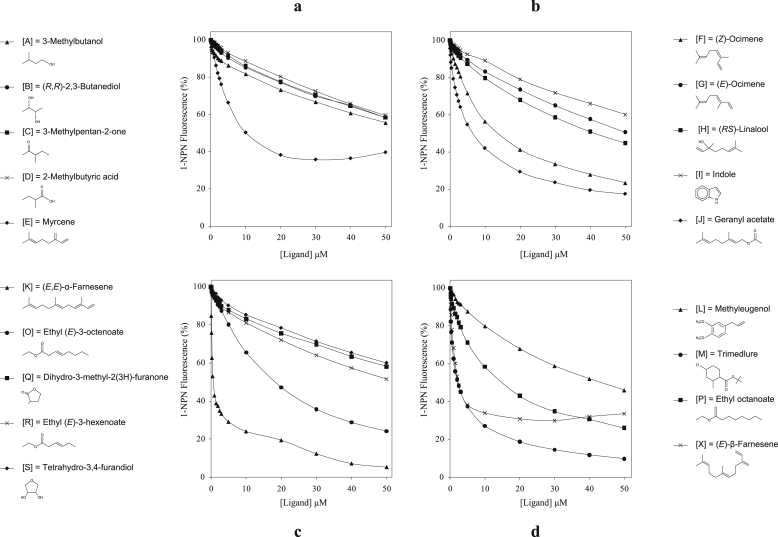
CcapOBP83a-2 and bioactive compounds binding results. The graphs report the relative fluorescence intensity of the CcapOBP83a-2/1-NPN complex at the ligand concentrations (μM). When the ligand concentration is 0, the complex fluorescence is represented as 100% and decreased when 1-NPN was displaced by a compound. The results for the 15 chemicals purified from the pheromone blend are represented in panels “a”, “b” and “c”. The panel “d” shows the binding results of three compounds (methyleugenol, Trimedlure and ethyl octanoate) and (*E*)-β-farnesene [as (*E*,*E*)-α-farnesene active isomer] previously demonstrated to have physiological/behavioural effect on the medfly.

**Fig. 10:**
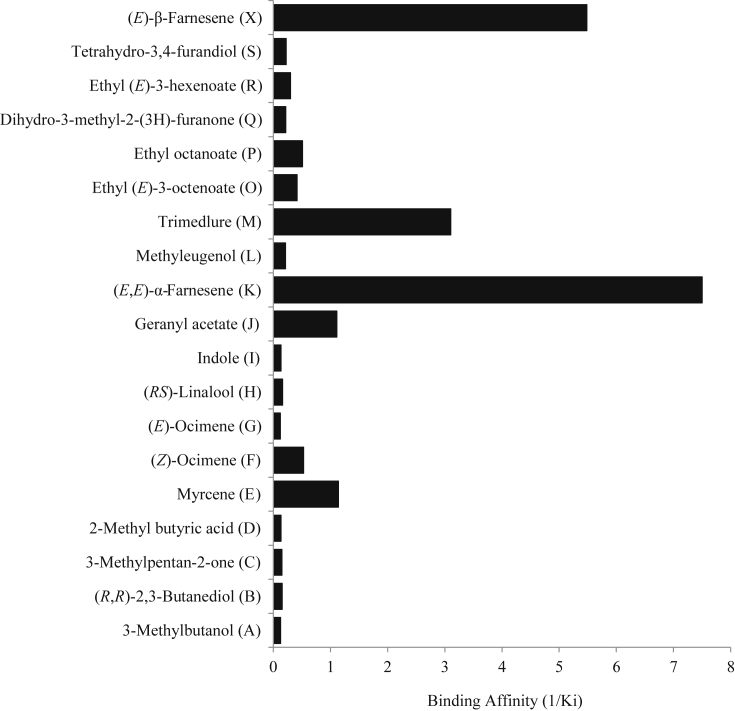
The binding affinity of each tested compound to CcapOBP83a-2. The affinity is reported as 1/Ki (Ki = dissociation constant calculated from data shown in Fig. 8). Only 5 chemicals [myrcene, geranyl acetate, (*E*,*E*)-α-farnesene and (*E*)-β-farnesene] displayed a binding affinity of 1/Ki > 1. The graph shows the best CcapOBP83a-2 binding affinity for (*E*,*E*)-α-farnesene and the ability of this protein to discriminate between isomers of the same chemical [(*E*,*E*)-α-farnesene and (*E*)-β-farnesene].

It is possible that the 1-NPN binding to the two CcapOBP83a-2 fractions is due to unspecific binding to the protein rather than within the binding pocket. It is also possible that the first fraction protein is aggregated and never properly refolded. We further investigated 1-NPN binding with Förster resonance energy transfer (FRET) analyses for the second fraction henceforth referred to as CcapOBP83a-2. CcapOBP83a-2 has a tryptophan residue near the predicted binding pocket and could cause FRET to the molecules bound in the pocket (He et al., 2010). Excitation of the tryptophan at 280 nm resulted in an energy transfer from the tryptophan residues to 1-NPN, as the concentration of 1-NPN increases the florescent emission of the 1-NPN/CcapOBP83a-2 complex at 410 nm increases, indicating an increase in 1-NPN binding to CcapOBP83a-2. However, at high concentrations (40 μM and 50 μM) 1-NPN caused unusual background and irregular binding curves (Fig. S5). The analyses indicated a possible specific binding of 1-NPN in the binding pocket of CcapOBP83a-2 (Fig. S5). We chose the second fraction to examine the binding affinity to OBP of electrophysiologically active compounds newly identified in present study. The results revealed a good binding affinity for: (*E*,*E*)-α-farnesene (Ki = 0.1 μM), (*E*)-β-farnesene (Ki = 0.2 μM), Trimedlure (Ki = 0.3 μM), geranyl acetate (Ki = 0.9 μM) and myrcene (Ki = 0.9 μM) (Fig. 9 and Fig. 10). The two ocimene isomers [(*Z*) and (*E*)] displayed very different binding affinities to CcapOBP83a-2: a low affinity (Ki = 1.9 μM) was detected for (*Z*)-ocimene, while there seemed to be no interaction between the peptide and (*E*) isomer (Ki = 8.2 μM), consist with the antennae EAG responses to these two ocimene isomer (Table 1). Also, ethyl octanoate displayed a low binding activity (Ki = 2.0 μM), while 11 compounds showed no affinity for CcapOBP83a-2 (Ki >> 1 μM).

The good level of binding affinity between CcapOBP83a-2 and electrophysiologically active compounds geranyl acetate and (*E*,*E*)-α-farnesene, as well as the behaviourally active synthetic molecule Trimedlure, reveals that CcapOBP83a-2 may be able to capture and transport these compounds to ORs. Trimedlure is known as a male attractant for the medfly and induced EAG response of both sexes with different affinities to the four trimedlure isomers (Jang et al., 1989). However, this fluorescent binding assay may not faithfully mimic what happens in the medfly antenna, where OBPs have to capture the chemicals from the air, and many other molecules in the antenna can interfere with the protein or with the chemical being studied. Also, temperature, pH and other environmental conditions are parameters that have to be considered in further analyses, since they could modulate OBP binding capacity by provoking slight structural changes in the binding pocket resulting in a modified binding affinity for a specific molecule. It is worthy to note that farnesene could also displace 1-NPN from other OBPs such as aphid OBP3 (Qiao et al., 2009), suggesting the binding of (*E*,*E*)-α-farnesene is not specific to CcapOBP83a-2. Nevertheless, the CcapOBP83a-2 binding affinity for (*E*,*E*)-α-farnesene (Ki = 0.1 μM) is much higher than for any other chemicals tested and leads to the assumption that (*E*,*E*)-α-farnesene may be the CcapOBP83a-2 ligands (or one of the many) in nature. Since (*E*,*E*)-α-farnesene is detected by both sexes (K in Fig. 1 and Table 1) and *CcapObp83a-2* transcript is mainly expressed in the antennae of both sexes and increased with sexual maturation (Hekmat-Scafe et al., 2000; Siciliano et al., 2014) it is not surprising that (*E*,*E*)-α-farnesene binds to CcapOBP83a-2 with a good affinity. The “farnesene family” refers to a group of six closely related sesquiterpenes. α-farnesene (3,7,11-trimethyl-1,3,6,10-dodecatetraene) and β-farnesene (7,11-dimethyl-3-methylene-1,6,10-dodecatriene) are isomers, differing only by the position of one of the double bonds. These farnesene stereoisomers have been demonstrated to be natural products [(*E*,*E*)-α-farnesene is the most common isomer] present in several plants such as green apple, gardenia and perilla (Wang et al., 2004), and as insect semiochemicals; for example, they act as alarm pheremones in termites (Sobotnik et al., 2008) and as food attractants for the apple tree pest *Cydia pomonella* (Hern and Dorn, 1999). This chemical has been demonstrated to be one of the five major components of the medfly pheromone blend (Fig. 1 and Table 1; also (Jang et al., 1989)) and an attractant of both sexes in different behavioural tests (Jang and Light, 1996). However, CcapOBP83a-2 binds to (*E*)-β-farnesene with similar affinity to (*E*,*E*)-α-farnesene, which shows its lower specificity for the position of the double bond in the farnesene molecules but some degree of ability to discriminate two isomers [(*Z*) and (*E*)] of the ocimene molecules with very different binding affinities.

## 4 Conclusions

Here we report the identification of pheromone components from the headspace of male medflies and their binding to a newly identified antenna enriched OBP, CcapOBP83a-2. Fifteen compounds with electrophysiological activity were identified in male emissions by air entrainment and gas chromatography coupled to electroantennography (GC–EAG). The binding studies with these bioactive compounds for the medfly revealed that CcapOBP83a-2 has binding affinity for some of the compounds tested, but not all. (*E*,*E*)-α-farnesene bound to CcapOBP83a-2 with much higher affinity than any other compounds tested. As (*E*,*E*)-α-farnesene is one of the major components of the medfly pheromone blend as determined here, this result suggests that this compound could be a natural ligand for CcapOBP83a-2. CcapOBP83a-2 affinity to other compounds, in particular to (*E*)-β-farnesene, revealed its low binding specificity. However, some degree of ability to discriminate between two isomers (ocimene) has been observed. Moreover, the binding ability of CcapOBP83a-2 to the synthetic molecule Trimedlure [t-Butyl-2-methyl-4-chlorocyclohexanecarboxylate], a strong attractant to the medfly, with relative high affinity suggests a possible role of CcapOBP83a-2 in the olfactory molecular pathway of the medfly. The amino acid sequence of CcapOBP83a-2 displays several common features with members of the insect PBP-GOBP superfamily (pfam01395), such as the presence of six cysteines in highly conserved positions, the presence of an expected-size signal peptide at the N-terminal and good levels of identity and similarity with OS-E and OS-F of pheromone sensitive sensilla expressing OBPs and with PBPs of other insect species including BmorPBP1, whose function as sex pheromone carrier has been demonstrated. These characteristics, together with the antennal enriched expression pattern of *CcapObp83a-2* gene (Siciliano et al., 2014), further suggest that CcapOBP83a-2 may play a role in the odour/pheromone perception in the medfly. Further analyses are needed in order to elucidate its function and demonstrate if this protein is indeed involved in the olfactory process *in vivo*, for example by RNA interfering approach to knock down its expression level in the antennae and electrophysiological and behavioural measurements of mutated flies. These also include the further analysis of CcapObp83a-1 of the OS-E/OS-F cluster.

Our study represents the first step in the clarification of the odour and pheromone perception pathway in this insect species and furnishes a very useful target for the design of synthetic attractants with higher binding strength and specificity, applicable in the field for the improvement of the current control techniques.

## Figures and Tables

**Table 1:** The fifteen electrophysiologically active compounds emitted by *C. capitata* males during their “calling” period. No chemical emissions were detected from females under the same experimental conditions (data not shown). Fourteen have already been reported in previous studies and tetrahydro-3,4-furandiol is a new compound. The last column indicates the numbers of males (M) and females (F) that showed a positive EAG response in a total of five replicates. The GC–EAG results are reported in Fig. 1.

Compound	Label	Mol. formula	EAG response
3-Methylbutan-1-ol	A	C_5_H_12_O	M4F4
(RR)-2,3-Butanediol	B	C_4_H_10_O_2_	M2F3
(RS)-3-Methylpentan-2-one	C	C_6_H_12_O	M0F3
(RS)-2-Methyl butyric acid	D	C_5_H_10_O_2_	M0F2
Dihydro-3-methyl-2-(3H)-furanone	Q	C_5_H_8_O_2_	M3F4
Tetrahydro-3,4-furandiol	S	C_4_H_8_O_3_	M2F4
Myrcene	E	C_10_H_16_	M0F4
Ethyl (E)-3-hexenoate	R	C_8_H_14_O_2_	M4F4
(Z)-Ocimene	F	C_10_H_16_	M4F4
(E)-Ocimene	G	C_10_H_16_	M0F4
(RS)-Linalool	H	C_10_H_18_O	M4F3
Ethyl (E)-3-octenoate	O	C_10_H_18_O_2_	M4F4
Indole	I	C_8_H_7_N	M3F3
Geranyl acetate	J	C_12_H_20_O_2_	M5F4
(E,E)-α-Farnesene	K	C_15_H_24_	M2F3

**Table 2:** The size (bp) and number of exons and interons in medfly OBPs and their *D. melenogaster* homologues.

OBP gene	GenBank id	Exon-1	Intron-1	Exon-2	Intron-2	Exon-3	Intron-3	Exon-4	Gene size
*CcapObp83a-1*	XM_004523388.1	123	207	76	5515	257	75	18	6271
*CcapObp83a-2*	XM_004523387.1	96	63	76	82	257	79	18	671
*DmelObp83a (OS–F)*	NM_079517.2	114	79	76	427	257	54	18	1025
*DmelObp83b (OS-E)*	NM_079518.2	72	62	76	51	278	Not apply	Not apply	539
