# Supplementary material for: Identification of pheromone components and their binding affinity to the odorant binding protein CcapOBP83a-2 of the Mediterranean fruit fly, Ceratitis capitata
Source: Insect Biochem Mol Biol. 2014 May;48(100):51–62. doi: 10.1016/j.ibmb.2014.02.005 (PMC4003389; doi:10.1016/j.ibmb.2014.02.005)
Supplement: Supplementary file 1 [file mmc1.pdf]

A. mRNA (699)
ACGTTAAACGAATTCCACCTCAACAGTGAATGTGGCAAGAGCAAGCACAATTTGACTAAAAAATAACC
AATTTAATTTACAGCTAAATAAAATGTATATCCTGAGAACCATTTTGGGTGCTTTGCTGTGGTGCAGC
GTTTTGCTGAATCTCATATGGGCACAAAAGGAGTTAAGACGTGATGAAACTTATCCTCCGCCGGAACT
GCTAGAGGCGCTCAGACCAGTACACGATAAGTGTGTTGCCAAAACCGGTGTTACAGAAGAGGCCATAA
AGGAGTTCAGTGATGGTGAGATACACGAAGATGAGCCACTCAAGTGCTATATGTACTGTGTGTTCGAA
GAAACGGATGTTTTGCATGAAGACGGCGAAGTGCATTTAGAGAAATTATTGGATAGTTTGCCGAACTC
AATGCACAACATCGCTTTGCATATGGGCAAGAAGTGTTTGTATCCCAAGGGTGACACGAAATGTGAAC
GCGCTTTTTGGTTACATCGCTGCTGGAAGGAGTCGGATCCAAAGCACTATTTCTTGATTTGAGAGCCG
CTTCATTGGAGGTCTTTCACAGCTGACTCTGCTTTTGACAATGTGAATACTGAACTTACATACATAAT
ATGTATTAATCTTTATTGTAGTCTTTTGGTTTATTATAATTTGCAAATATAGTTTAACGAACATTCCA
AAAAAAAAAAAAAAAAAAA

B. CDS (447)
ATGTATATCCTGAGAACCATTTTGGGTGCTTTGCTGTGGTGCAGCGTTTTGCTGAATCTCATATGGGC
ACAAAAGGAGTTAAGACGTGATGAAACTTATCCTCCGCCGGAACTGCTAGAGGCGCTCAGACCAGTAC
ACGATAAGTGTGTTGCCAAAACCGGTGTTACAGAAGAGGCCATAAAGGAGTTCAGTGATGGTGAGATA
CACGAAGATGAGCCACTCAAGTGCTATATGTACTGTGTGTTCGAAGAAACGGATGTTTTGCATGAAGA
CGGCGAAGTGCATTTAGAGAAATTATTGGATAGTTTGCCGAACTCAATGCACAACATCGCTTTGCATA
TGGGCAAGAAGTGTTTGTATCCCAAGGGTGACACGAAATGTGAACGCGCTTTTTGGTTACATCGCTGC
TGGAAGGAGTCGGATCCAAAGCACTATTTCTTGATTTGA

C. Amino acid (148 aa – 17.4 Kdalton)
MYILRTILGALLWCSVLLNLIWAQKELRRDETYPPPELLEALRPVHDKCVAKTGVTEEAIKEFSDGEI
HEDEPLKCYMYCVFEETDVLHEDGEVHLEKLLDSLPNSMHNIALHMGKKCLYPKGDTKCERAFWLHRC
WKESDPKHYFLI
