# Supplementary figures and images for: Identification of pheromone components and their binding affinity to the odorant binding protein CcapOBP83a-2 of the Mediterranean fruit fly, Ceratitis capitata
Source: Insect Biochem Mol Biol. 2014 May;48(100):51–62. doi: 10.1016/j.ibmb.2014.02.005 (PMC4003389; doi:10.1016/j.ibmb.2014.02.005)

Supplementary Figure S2

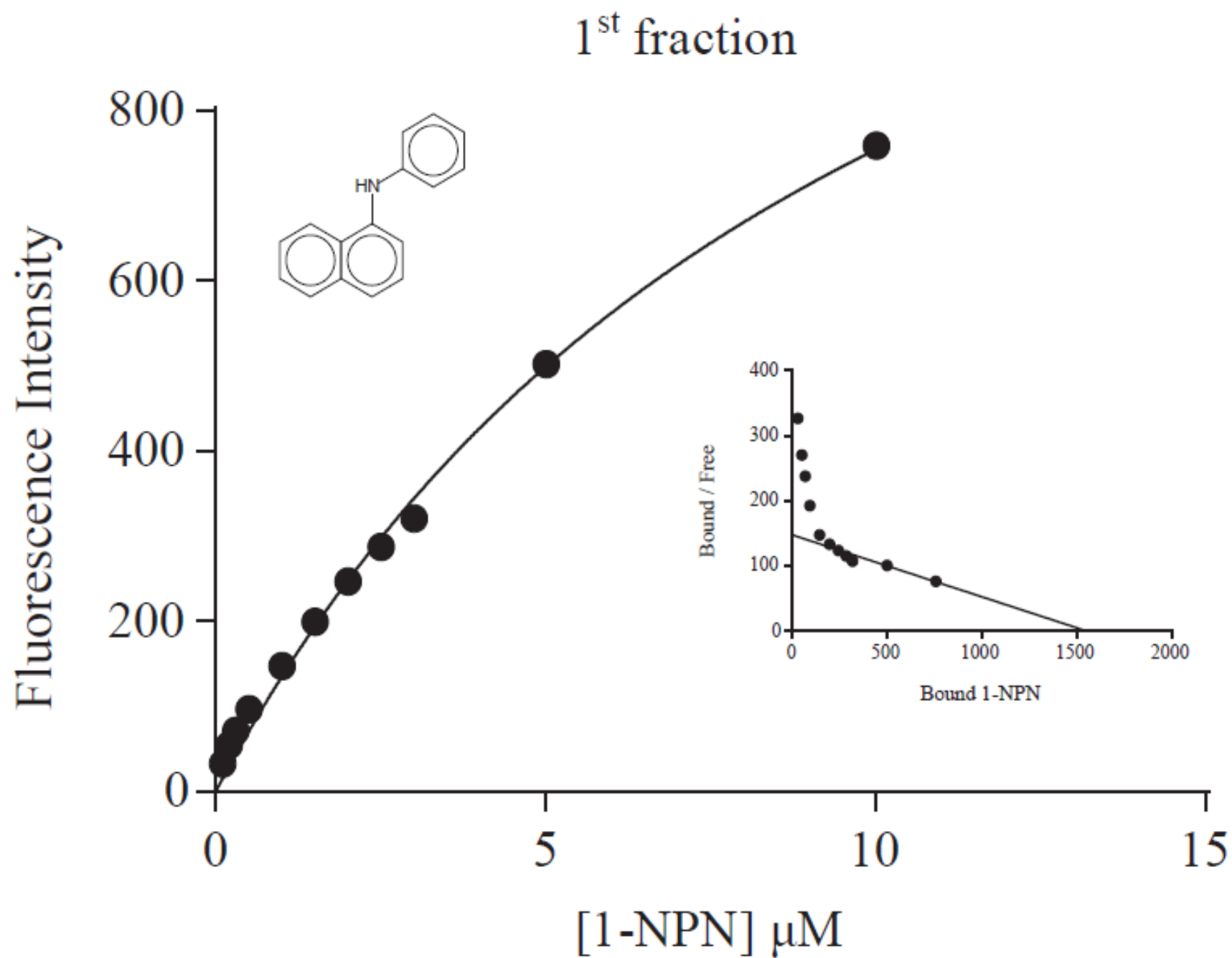

Supplement: Supplementary file 2 [file mmc2.pdf]

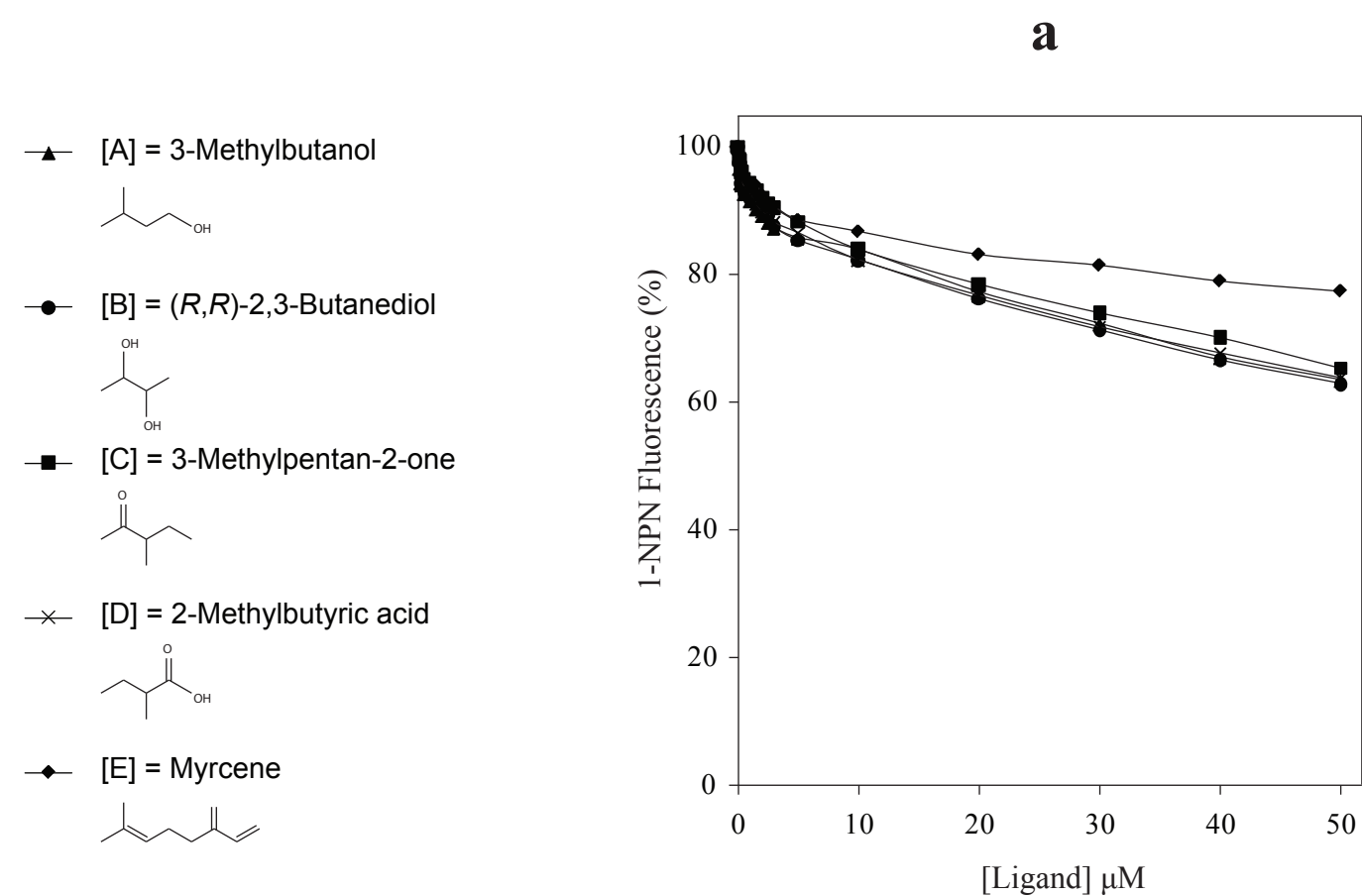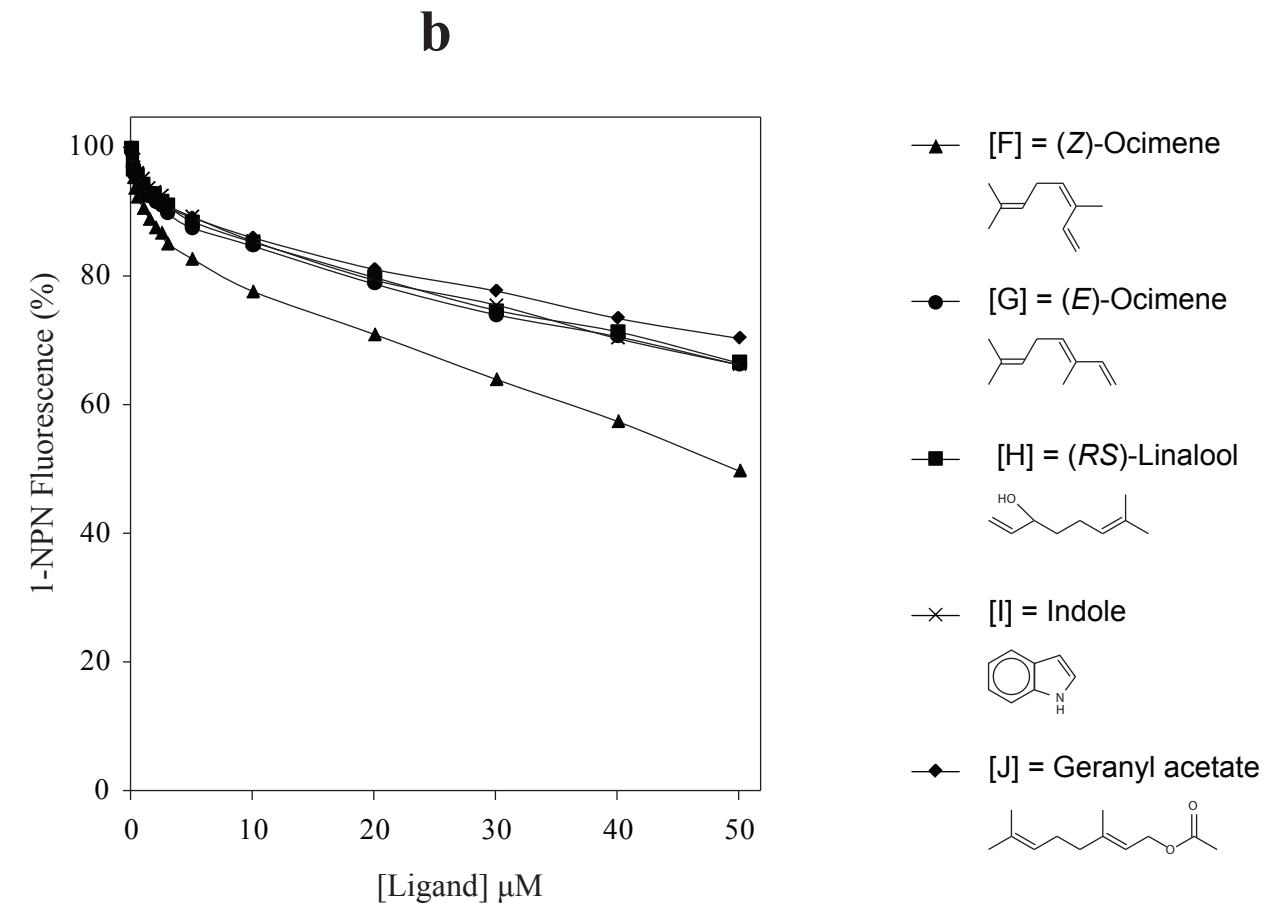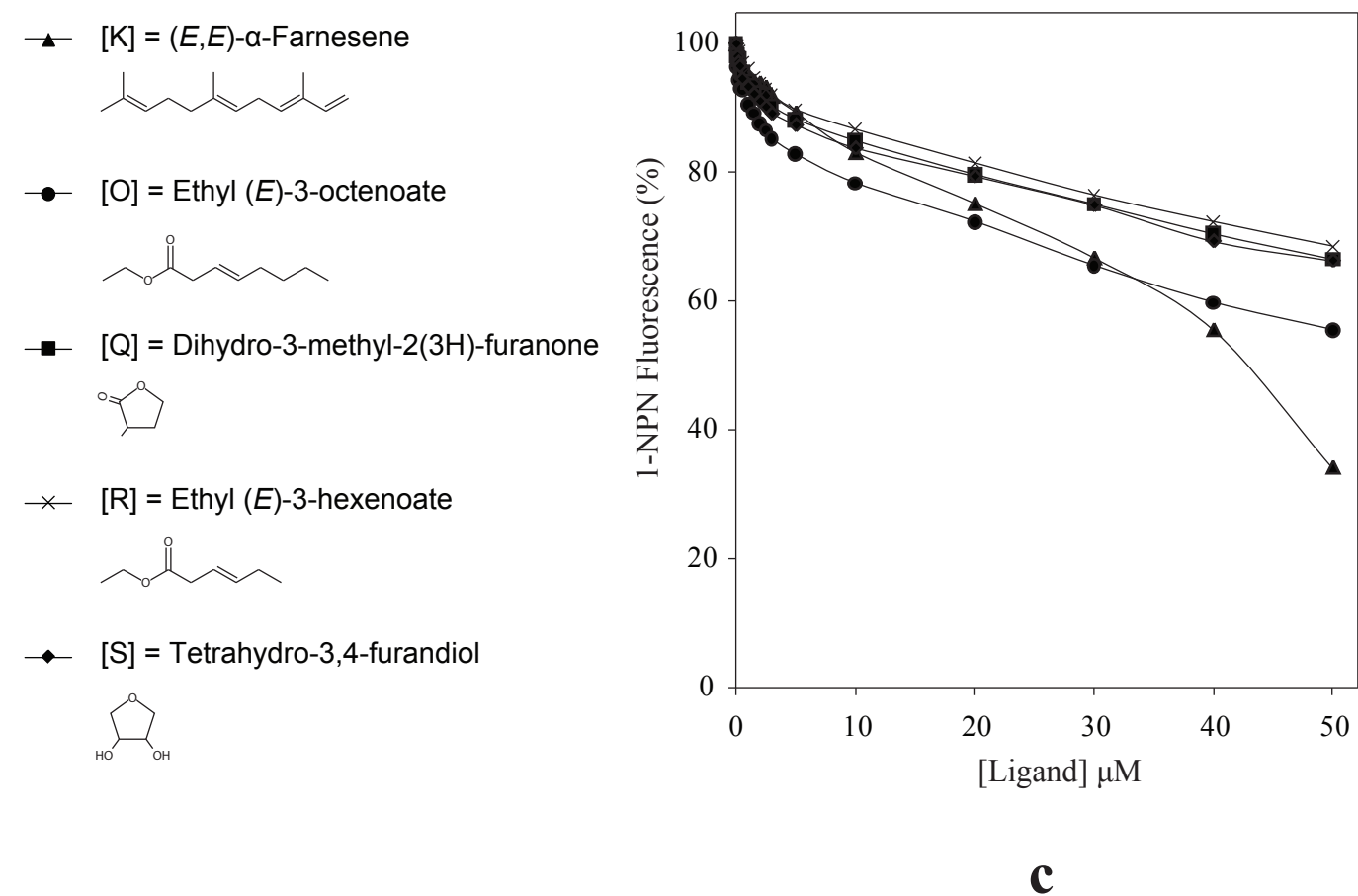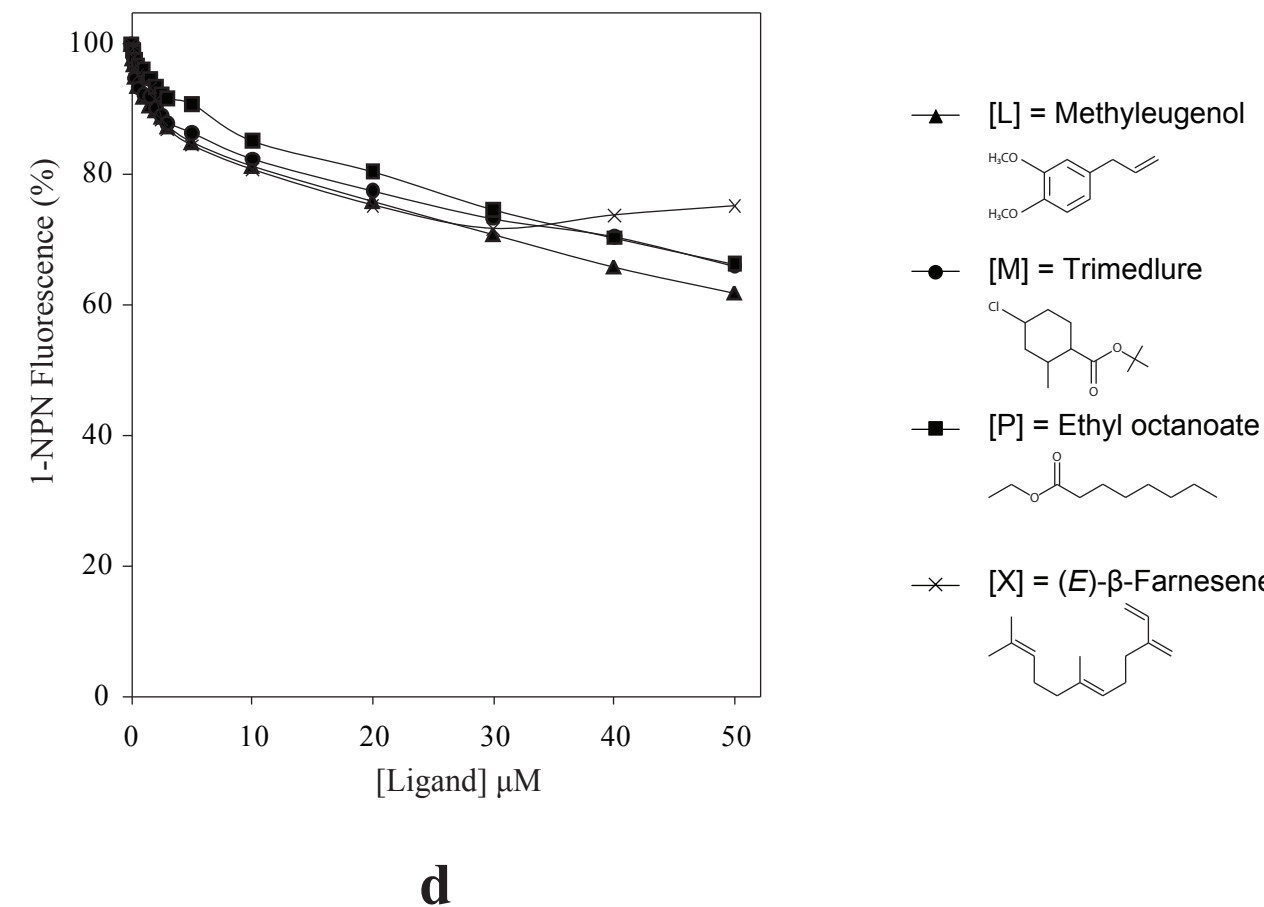

Supplement: Supplementary file 3 [file mmc3.pdf]

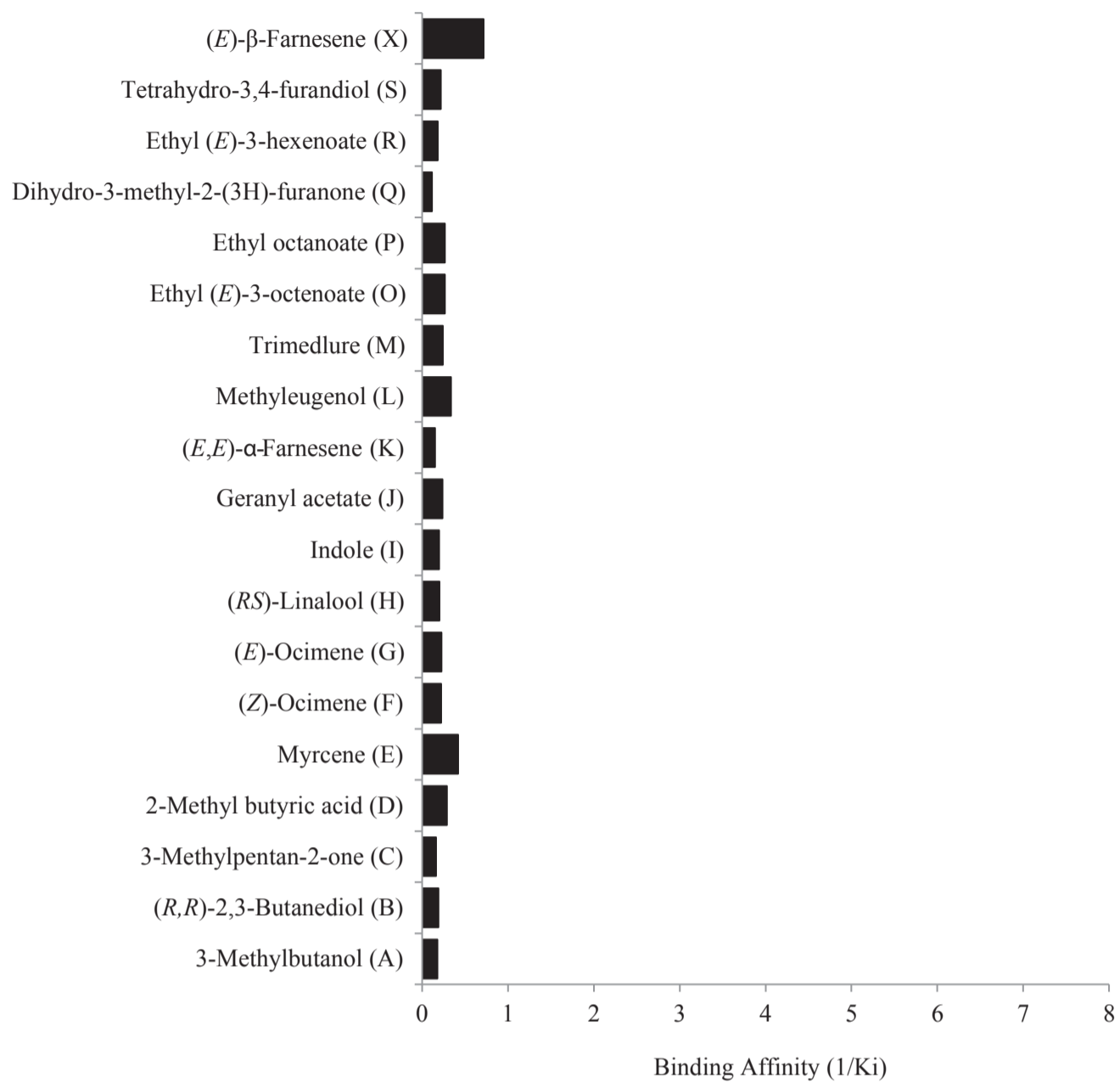

Supplement: Supplementary file 4 [file mmc4.pdf]

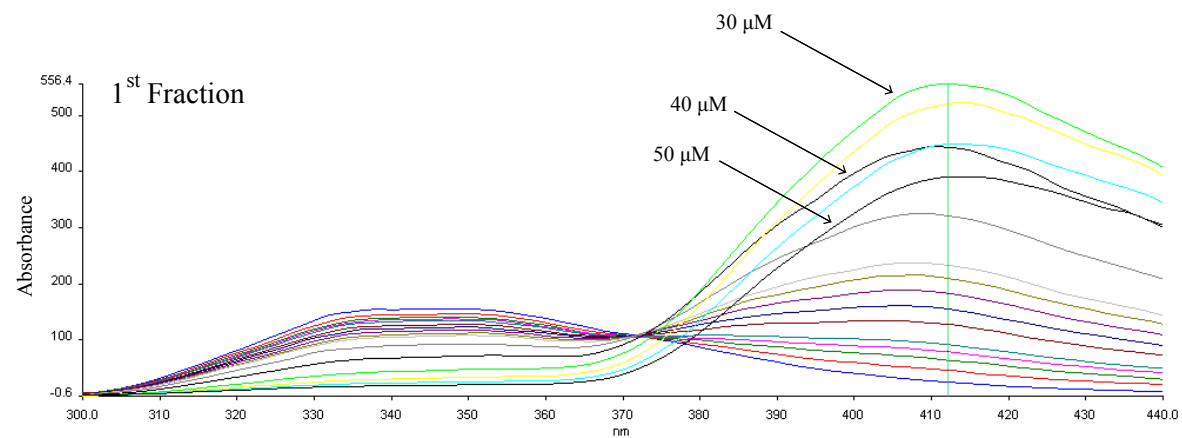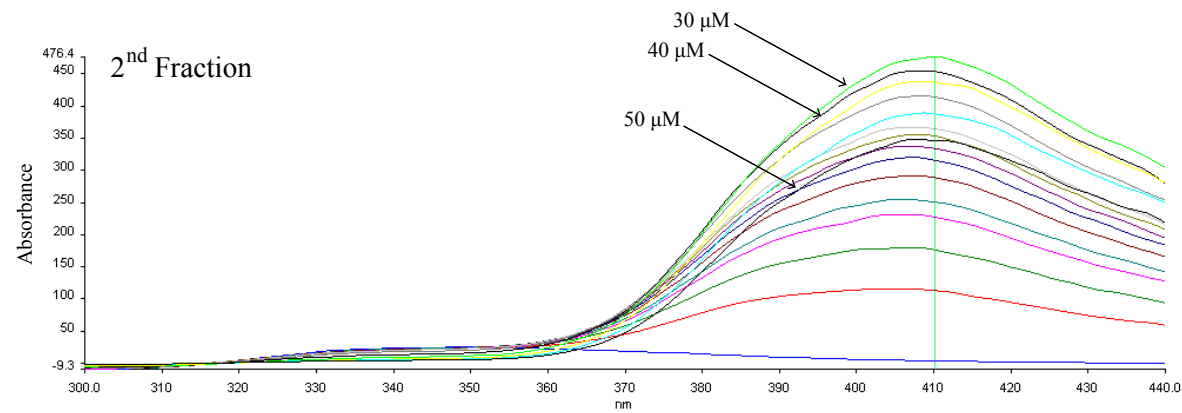

Supplement: Supplementary file 5 [file mmc5.pdf]
