# Supplementary material for: Identification of pheromone components and their binding affinity to the odorant binding protein CcapOBP83a-2 of the Mediterranean fruit fly, Ceratitis capitata
Source: Insect Biochem Mol Biol. 2014 May;48(100):51–62. doi: 10.1016/j.ibmb.2014.02.005 (PMC4003389; doi:10.1016/j.ibmb.2014.02.005)
Supplement: Supplementary file 6 [file mmc6.docx]

***Supplementary***

**Table S1.** Identity and similarity between CcapOBP83a-2 and other known insect PBP-like proteins.

| *Protein* | *Identity* | *Similarity* |
| --- | --- | --- |
| BdorOBP | 83.78% | 88.51% |
| GmorOBP8 | 34.00% | 44.59% |
| TcasOBP6 | 11.27% | 18.24% |
| AgamOBP17 | 11.11% | 17.56% |
| AmelPBP-ASP1 | 9.72% | 18.24% |
| MsexPBP1 | 9.31% | 20.94% |
| DmPbprp1 | 8.78% | 19.59% |
| DmPbprp3 | 8.44% | 20.94% |
| MdomOBP3 | 8.44% | 21.62% |
| DmPbprp5 | 8.39% | 16.89% |
| TcasOBP8 | 8.27% | 14.86% |
| DmPbprp2 | 8.00% | 15.54% |
| BmorPBP1 | 7.92% | 16.89% |
| CpipOBP1 | 7.38% | 18.91% |
| AalbOBP2 | 6.99% | 17.56% |
| AaegOBP1 | 6.29% | 17.56% |
| MsexABP3 | 5.67% | 11.48% |
| LUSH | 5.22% | 16.21% |
| DantOBP2 | 4.66% | 13.51% |
| DmPbprp4-PA | 4.57% | 14.86% |
| DmPbprp4-PC | 4.52% | 16.89% |

**Table S2.** First fraction binding results. IC50: concentration of ligand halving the initial fluorescence level; Int (end point fluorescent level): fluorescence intensity after the maximum quantity of chemical was added (i.e. [50μM]); Ki: Dissociation constant.

| Ligand | Ref Letter | IC50 (μM) | Int | Ki | 1/Ki |
| --- | --- | --- | --- | --- | --- |
| 3-Methylbutanol | A | - | 63.4 | 5.594 | 0.179 |
| (*R*,*R*)-2,3-Butanediol | B | - | 62.9 | 5.230 | 0.191 |
| 3-Methylpentan-2-one | C | - | 65.3 | 6.205 | 0.161 |
| 2-Methyl butyric acid | D | - | 63.7 | 3.456 | 0.289 |
| Myrcene | E | - | 77.5 | 2.382 | 0.420 |
| (*Z*)-Ocimene | F | - | 49.9 | 4.516 | 0.221 |
| (*E*)-Ocimene | G | - | 66.3 | 4.383 | 0.228 |
| (*RS*)-Linalool | H | - | 66.6 | 4.892 | 0.204 |
| Indole | I | - | 66.2 | 4.992 | 0.200 |
| Geranyl acetate | J | - | 70.4 | 4.223 | 0.237 |
| (*E*,*E*)-α-Farnesene | K | - | 34.3 | 6.564 | 0.152 |
| Methyleugenol | L | - | 61.9 | 2.981 | 0.335 |
| Trimedlure | M | - | 66.0 | 4.144 | 0.241 |
| Ethyl (*E*)-3-octenoate | O | - | 55.6 | 3.739 | 0.267 |
| Ethyl octanoate | P | - | 66.3 | 3.739 | 0.267 |
| Dihydro-3-methyl-2-(3H)-furanone | Q | - | 66.5 | 8.699 | 0.115 |
| Ethyl (*E*)-3-hexenoate | R | - | 68.5 | 5.515 | 0.181 |
| Tetrahydro-3,4-furandiol | S | - | 66.2 | 4.544 | 0.220 |
| (*E*)-β-Farnesene | X | - | 75.1 | 1.397 | 0.720 |

**Table S3.** Second fraction binding results. IC50: concentration of ligand halving the initial fluorescence level; Int (end point fluorescent level): fluorescence intensity after the maximum quantity of chemical was added (i.e. [50μM]); Ki: Dissociation constant.

| Ligand | Ref Letter | IC50 (μM) | Int | Ki | 1/Ki |
| --- | --- | --- | --- | --- | --- |
| 3-Methylbutanol | A | - | 55.6 | 7.935 | 0.126 |
| (*R*,*R*)-2,3-Butanediol | B | - | 58.3 | 6.372 | 0.157 |
| 3-Methylpentan-2-one | C | - | 58.3 | 6.604 | 0.151 |
| 2-Methyl butyric acid | D | - | 59.6 | 7.488 | 0.134 |
| Myrcene | E | 10 | 39.6 | 0.879 | 1.138 |
| (*Z*)-Ocimene | F | 13.5 | 23.4 | 1.888 | 0.530 |
| (*E*)-Ocimene | G | 50 | 50.7 | 8.173 | 0.122 |
| (*RS*)-Linalool | H | 41 | 44.7 | 6.096 | 0.164 |
| Indole | I | - | 60.1 | 7.305 | 0.137 |
| Geranyl acetate | J | 6.5 | 17.4 | 0.901 | 1.110 |
| (*E*,*E*)-α-Farnesene | K | 1.2 | 14.4 | 0.133 | 7.502 |
| Methyleugenol | L | 43 | 46.0 | 4.638 | 0.216 |
| Trimedlure | M | 2.1 | 9.8 | 0.322 | 3.105 |
| Ethyl (*E*)-3-octenoate | O | 18 | 24.2 | 2.408 | 0.415 |
| Ethyl octanoate | P | 15 | 26.1 | 1.964 | 0.509 |
| Dihydro-3-methyl-2-(3H)-furanone | Q | - | 58.1 | 4.589 | 0.218 |
| Ethyl (*E*)-3-hexenoate | R | - | 51.5 | 3.297 | 0.303 |
| Tetrahydro-3,4-furandiol | S | - | 59.9 | 4.439 | 0.230 |
| (*E*)-β-Farnesene | X | - | 33.6 | 0.182 | 5.490 |

**Figure S1.** *CcapObp83a-2* mRNA (a) and CDS (b) nucleotide sequences and CcapOBP83a-2 amino acid sequence (c). Underlined and in bold are represented the nucleotide (b) and amino acids (c) composing the signal peptide.

**Figure S2.** Binding of 1-NPN to first fraction CcapOBP83a-2. The purified fraction CcapOBP83a-2 was diluted in 20 mM Tris-HCl pH 7.4 with a final concentration of 2 μM. Aliquots of 1-NPN stock solution [1mM] in methanol were added to a final concentration of 0.1, 0.2, 0.3, 0.5, 1, 1.5, 2, 2.5, 3, 5 and 10 μM. Excitation was set at 337 nm, the peak emission at 380-460 nm was recorded and plotted against 1-NPN concentrations. The curve was used to determine the dissociation constant (K_D_ 1^st^ fraction: 10.5±1.4 µM) by nonlinear regression curve fitting using GraphPad Prism 5, and transformed to Scatchard plot (insert).

**Figure S3.** First fraction binding assay results. The graphs report the fluorescence level of the peptide/1-NPN complex on the ligand concentration (expressed in µM). When the ligand concentration is 0, the complex fluorescence is represented as 100%. As reported in Figure 7, the results for the 15 chemicals purified from the pheromone blend are represented in panels “a”, “b” and “c”. In the panel “d”, we report the binding results relative to three compounds previously demonstrated to have physiological/behavioural effects on the medfly (methyleugenol, Trimedlure and ethyl octanoate) and (*E*)-β-farnesene [as (*E*,*E*)-α-farnesene active isomer].

**Figure S4.** First fraction binding affinity for each tested compound is reported in 1/Ki (Ki = dissociation constant). The peptide displays no binding affinity for any of the tested compounds (1/Ki < 1 in each test).

**Figure S5.** Fӧrster Resonance Energy Tranfer (FRET) on 1^st^ and 2^nd^ fraction peptides. Excitation: 280 nm; range emission: 300-450 nm; protein [2µM] in 20 mM Tris-HCl pH 7.4; 1-NPN [0, 0.1, 0.2, 0.3, 0.5, 1, 1.5, 2, 2.5, 3, 5, 10, 20, 30, 40 and 50 µM]. The energy deriving from tryptophan residues excitation is successfully transferred to the 1-NPN in the binding pocket in both the fractions. Protein emission range: 330-350 nm; protein/1-NPN complex emission range: 405-415 nm. Saturation is reached at 1-NPN [30 µM] for both peptides. [40 µM] and [50 µM] 1-NPN are out of scale. The green vertical line represents the abscissa at which the maximum fluorescence is read.
